# Paternity leave, supportive involvement, and maternal postpartum adjustment: parity-stratified associations in a structural equation model

**DOI:** 10.3389/fpsyg.2026.1807103

**Published:** 2026-07-14

**Authors:** Aiko Hironaka, Shoko Sugao, Masayuki Endo, Hiroko Watanabe

**Affiliations:** 1 Faculty of Health Science, Okayama University, Okayama, Japan; 2 Department of Children and Women’s Health, The University of Osaka Graduate School of Medicine, Suita, Japan; 3 Department of Clinical Psychology, Graduate School of Human Sciences, The University of Osaka, Suita, Japan; 4 Department of Experimental Psychology, University of Oxford, Oxford, United Kingdom; 5 Department of Obstetrics and Gynecology, Graduate School of Medicine, The University of Osaka, Suita, Japan

**Keywords:** paternity leave, coparenting, maternal depressive symptoms, parenting resilience, postpartum adjustment, parity, structural equation modeling

## Abstract

**Background:**

How paternity leave relates to postpartum adjustment remains unclear.

**Objective:**

We examined associations of paternity leave group [no leave, short (<1 month), or long (≥1 month)] with mothers’ parenting adaptation and depressive symptoms at 3 months postpartum in a theory-guided SEM including mothers’ reports of fathers’ childcare and housework involvement and perceived coparenting quality.

**Methods:**

This cross-sectional survey included 555 mothers (primipara, *n* = 330; multipara, *n* = 225). Participants completed questionnaires assessing fathers’ involvement using the Child Care Activities and Housework Scale, coparenting quality using the Coparenting Relationship Scale, parenting resilience and adaptation using the Comprehensive Scale for Parenting Resilience and Adaptation, and depressive symptoms using the Center for Epidemiologic Studies Depression Scale. Parity-stratified SEM estimated associations.

**Results:**

In primiparous families, short and long leave were associated with higher paternal involvement; long leave had stronger associations across all four domains: Interactional Activities, Concrete Childcare Activities, Mental Support, and Housework. In multiparous families, leave was associated mainly with housework and task-based childcare, but not clearly with mental support. Across parity groups, mothers’ reports of fathers’ mental support showed the strongest association with mothers’ perceived coparenting quality [primipara: *β* = 0.411, 95% CI (0.285, 0.527); multipara: *β* = 0.490, 95% CI (0.350, 0.612)]. Better coparenting was associated with fewer perceived difficulties in environmental resources and perceived support and with lower psychological maladaptation to parenting; psychological maladaptation was positively associated with depressive symptoms in both groups [primipara: *β* = 0.396, 95% CI (0.178, 0.571); multipara: *β* = 0.301, 95% CI (0.133, 0.459)]. Because no direct paths from paternity leave to depressive symptoms were specified, total effects represented indirect associations. These associations were small and negative for both short and long leave among primiparous mothers; among multiparous mothers, only short leave showed a small negative indirect association, whereas the association for long leave was not clearly supported.

**Conclusion:**

Paternity leave duration showed small indirect associations with maternal parenting adaptation and depressive symptoms through fathers’ involvement and coparenting quality. The findings suggest that the potential relevance of leave may depend not only on uptake and duration but also on supportive involvement and coparenting.

## 1 Introduction

Promoting fathers’ uptake of paternity leave is attracting increasing global attention as a policy measure to advance gender equality and support families in the context of low fertility. Childbirth and early childrearing are closely tied to persistent gender gaps in labor-market outcomes across many societies and are often described as “child penalties” (Angelov et al., 2016; Kleven et al., 2019). Furthermore, cross-national research suggests that family-policy design and the broader institutional and normative context can shape how paid work and unpaid care are divided within households, with consequential implications for gender inequality and family well-being (Olivetti and Petrongolo, 2017). Therefore, encouraging fathers’ leave-taking can promote a more equitable division of unpaid work and potentially reduce maternal burden during the postpartum period.

Recent legal reforms have rapidly increased paternity leave uptake in Japan (Ministry of Health, Labour and Welfare, 2023). However, a critical gap remains among leave uptake, leave duration, and the extent to which leave-taking is associated with fathers’ actual childcare, housework, and supportive behaviors. Considering Japan’s large gender gap in unpaid work relative to other OECD countries (OECD, 2024), clarifying whether paternity leave is associated with meaningful changes in fathers’ domestic roles and family functioning is important. Evidence from policy reforms in other settings also indicates that policy design can affect whether leave uptake is accompanied by measurable changes in childcare and housework (Patnaik, 2019). Therefore, treating leave-taking as a simple yes/no indicator may be inadequate; examining leave duration together with fathers’ actual involvement is important. Accordingly, we distinguished whether leave was taken, how long it was taken, and mothers’ reports of fathers’ involvement at 3 months postpartum. Previous research on paternity leave has yielded mixed findings. Some studies have suggested that leave-taking is associated with improved relationship quality, father engagement, and coparenting (Knoester et al., 2019; Petts and Knoester, 2018, 2020; Yeung and Li, 2023). However, others suggest that it does not uniformly translate into positive family processes. Furthermore, cross-national evidence indicates that fathers availing parental leave may maintain higher levels of childcare and housework involvement even after returning to work (Bünning, 2015). However, a Japanese study reported that fathers’ paternity leave was associated with greater impairment in father–infant bonding, particularly higher anger and rejection (Terada et al., 2022). Moreover, literature highlights heterogeneity in how leave is experienced and used, shaped by parental preferences, employer support, and anticipated work–family conflict (Schaber et al., 2021). These inconsistencies underscore the importance of examining whether paternity leave is linked to paternal involvement (e.g., interactive caregiving, hands-on childcare, mental support for mothers, and housework), and whether such involvement fosters a further collaborative coparenting relationship.

In the Japanese context, paternity leave may not uniformly correspond to favorable family processes. The adverse association with father–infant bonding reported by Terada et al. (2022) may reflect several nonexclusive explanations. Policy design and leave duration may determine whether leave provides sufficient opportunity for sustained caregiving or instead creates a brief and stressful transition. Limited workplace support, concern about burdening colleagues, and anticipated career disadvantage may increase guilt or work–family conflict during leave. Fathers may also begin leave without adequate caregiving knowledge, confidence, or social support, and their own psychological adjustment may shape both how leave is experienced and how they engage with the family. In addition, self-selection is possible because workplace flexibility, pre-existing family difficulties, and fathers’ psychological characteristics may influence both leave-taking and family outcomes. Accordingly, we did not posit a uniformly beneficial direct association between leave and maternal outcomes; instead, the model examined leave group in relation to mothers’ reports of fathers’ involvement and perceived coparenting quality.

Coparenting refers to parents’ ability to work together effectively as a parenting team; it involves coordination and mutual support in childrearing, and is an important family process linked to parents’ adjustment and mental health (De Palma et al., 2023; Feinberg, 2003). In Japan, higher coparenting quality has been associated with lower maternal depressive symptoms and less negative mother–infant bonding (Takeishi et al., 2021). Furthermore, prenatal couple-based education improved coparenting and reduced parenting stress and childbirth anxiety (Takeishi et al., 2019). Recent systematic reviews have further indicated that paternity leave effects vary according to context and measurement, highlighting the need for mechanism-focused models (Pizarro and Gartzia, 2024). Together, these mixed findings underscore the importance of examining fathers’ specific involvement and coparenting rather than assuming uniformly favorable associations of leave-taking with family outcomes. Thus, fathers’ actual involvement may help clarify when and how paternity leave is associated with maternal postpartum adjustment. Paternal involvement and coparenting are related but conceptually distinct constructs. Paternal involvement refers to fathers’ specific childcare, housework, and supportive behaviors, whereas coparenting refers to the dyadic relational process through which parents coordinate, support, and negotiate parenting as a team. Within the paternal-involvement measure, Mental Support captures specific father-to-mother supportive behaviors, such as listening to the mother’s concerns. By contrast, the CRS captures mothers’ perceptions of the broader dyadic parenting-team relationship across agreement, closeness, support, undermining, conflict exposure, endorsement of the partner’s parenting, and division of labor (Feinberg et al., 2012). Thus, Mental Support is a specific behavioral domain, whereas coparenting is a multidimensional relational construct. Fathers’ day-to-day involvement may provide a behavioral context that is associated with higher coparenting quality, particularly when involvement includes emotional support and shared planning.

Therefore, focusing solely on postpartum depressive symptoms may be insufficient to comprehensively capture maternal adjustment. Recent research in Japan emphasizes that adaptation to child-rearing is shaped by multiple environmental and individual factors; therefore, a comprehensive assessment is required to prevent parenting maladjustment and provide targeted support (Sugao et al., 2022). Similarly, literature highlights that maternal adaptation and adjustment are influenced by intersecting contextual resources and individual characteristics, including among working mothers (Ahn et al., 2021). Hence, coparenting can be conceptualized as an interpersonal resource that may buffer stress and enhance mothers’ perceived resources and adaptation in parenting.

The ordering of variables integrated three complementary sources rather than a single causal theory. First, paternity-leave research suggests that leave can provide time and institutional opportunity associated with fathers’ childcare and housework involvement (Bünning, 2015; Patnaik, 2019; Petts and Knoester, 2018). Second, Feinberg’s ecological model conceptualizes coparenting as a family subsystem embedded in contextual and parent-level influences and linked to parental adjustment (Feinberg, 2003). Third, the CPRA framework distinguishes Environmental Resources, Perceived Support, Mother’s Cognitive and Behavioral Characteristics, and Psychological Adaptation to Parenting and documents associations of these domains with depressive symptoms (Sugao et al., 2022). On this basis, the SEM ordered paternity leave group, mothers’ reports of fathers’ involvement, mothers’ perceived coparenting quality, CPRA domains, and depressive symptoms. Because all variables were assessed at the same time, this ordering was used to organize contemporaneous associations and estimate indirect associations; it does not establish temporal or causal precedence.

In our model, coparenting was conceptualized as an interpersonal resource associated with mothers’ perceived environmental resources, perceived support, and parenting-related adaptation. Higher coparenting quality may be associated with fewer perceived difficulties in resilience-related domains and lower psychological maladaptation to parenting, which may in turn be associated with fewer depressive symptoms. Further, maternal adaptation to parenting and depressive symptoms are related but distinct aspects of early postpartum adjustment. Maternal adaptation reflects parenting-related resources, perceived difficulties, and maladaptation in the caregiving context, whereas depressive symptoms represent broader mental health symptom burden. Therefore, examining both constructs may provide a more comprehensive understanding of how family processes are associated with mothers’ early postpartum adjustment. In our model, maternal parenting adaptation was positioned as a proximal parenting-related correlate of depressive symptoms. Difficulties in parenting resources and psychological maladaptation may co-occur with, and theoretically contribute to, depressive symptom severity. However, because the present study is cross-sectional, this sequence should be interpreted as a theoretically specified association rather than evidence of temporal or causal ordering, as with coparenting and depressive symptoms.

Despite increasing policy emphasis on paternity leave, few studies have assessed this serial process—from paternity leave, via paternal behavioral involvement and coparenting, to maternal resilience-related resources/adaptation and mental health—within a single analytic framework in Japan. First-time parents are developing new parenting roles, routines, and coparenting patterns during the transition to parenthood, whereas multiparous families may have established routines but face additional demands related to older-child care, time constraints, and household management. These contextual differences motivated parity-stratified estimation to describe group-specific patterns. Parity was treated as a prespecified stratification factor rather than as a formal moderator, and the study did not test whether individual structural coefficients differed significantly between groups.

**Figure 1:**
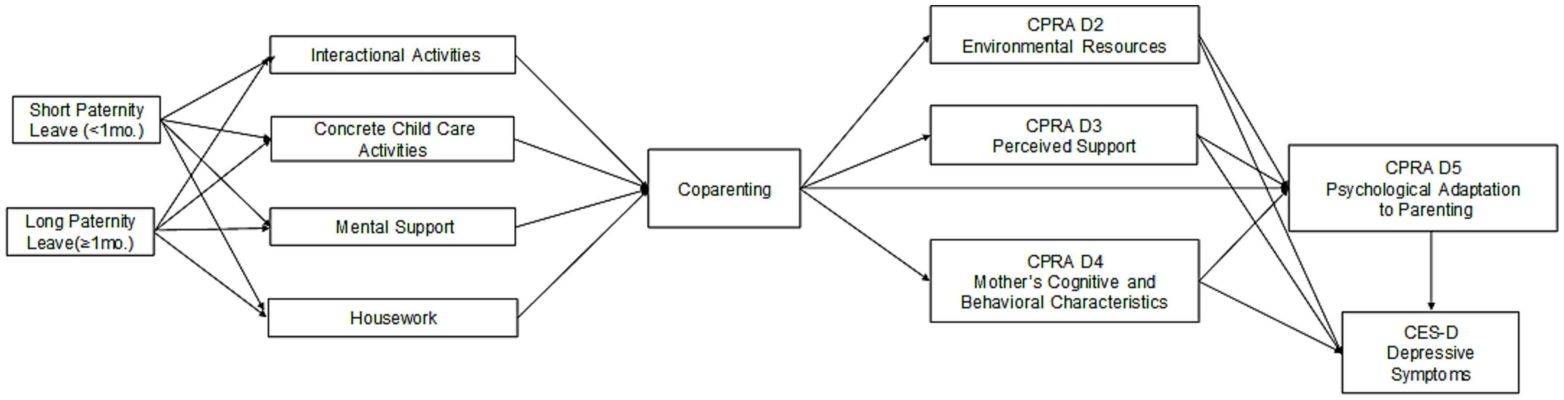
Conceptual model of the hypothesized associations among paternity leave duration, paternal involvement, coparenting quality, maternal parenting adaptation, and depressive symptoms. Short and long paternity leave were modeled as dummy variables, with the no-leave group as the reference category. Paternal involvement included four observed domains: Interactional Activities, Concrete Childcare Activities, Mental Support, and Housework. Maternal parenting resilience/adaptation was represented by CPRA D2–D5. Higher CPRA scores indicate greater parenting-related difficulties or poorer adaptation. The model was estimated separately for primiparous and multiparous mothers using multi-group SEM. Covariates are omitted for clarity. Because the study was cross-sectional, arrows represent theoretically specified associations rather than causal effects.

This study examined whether fathers’ paternity leave duration (short vs. long compared with no leave) was associated with mothers’ psychological adaptation to parenting and depressive symptoms at 3 months postpartum, and whether these associations were linked to fathers’ childcare/housework involvement and coparenting quality. Furthermore, we aimed to estimate these associations separately for primiparous and multiparous mothers and to describe parity-stratified, group-specific patterns using multi-group structural equation modeling (SEM). Accordingly, we hypothesized that: *H1*: Fathers’ paternity leave (short and long) would be associated with higher paternal involvement in childcare and housework behaviors. Furthermore, these behaviors would be positively associated with coparenting quality. *H2*: Higher coparenting quality would be associated with fewer perceived difficulties in maternal parenting resilience resources and better psychological adaptation to parenting, which would be associated with fewer depressive symptoms. Figure 1 illustrates the conceptual model of the hypothesized associations.

## 2 Materials and methods

### 2.1 Participants and procedure

Data were obtained via an online survey conducted from May to September 2025. Participants were recruited through two channels: an online research company (Freeasy; iBRIDGE Corporation, Osaka, Japan) and email/LINE announcements distributed by a baby-product company. We targeted mothers rearing a singleton infant at 3 months postpartum. The inclusion criteria were: (1) ability to complete the questionnaire in Japanese and (2) cohabitation with the child’s father. Before enrollment, participants completed a self-reported screening item regarding previous diagnoses of listed mental disorders, and those reporting any listed diagnosis were excluded. Postpartum depression was not assessed as a separate diagnostic category or used as a separate exclusion criterion. However, a self-reported diagnosis of postpartum depression would have met the exclusion criterion under the broader category of depression. Participants were not excluded on the basis of their CES-D scores, and no clinical diagnostic interview was conducted.

**Figure 2:**
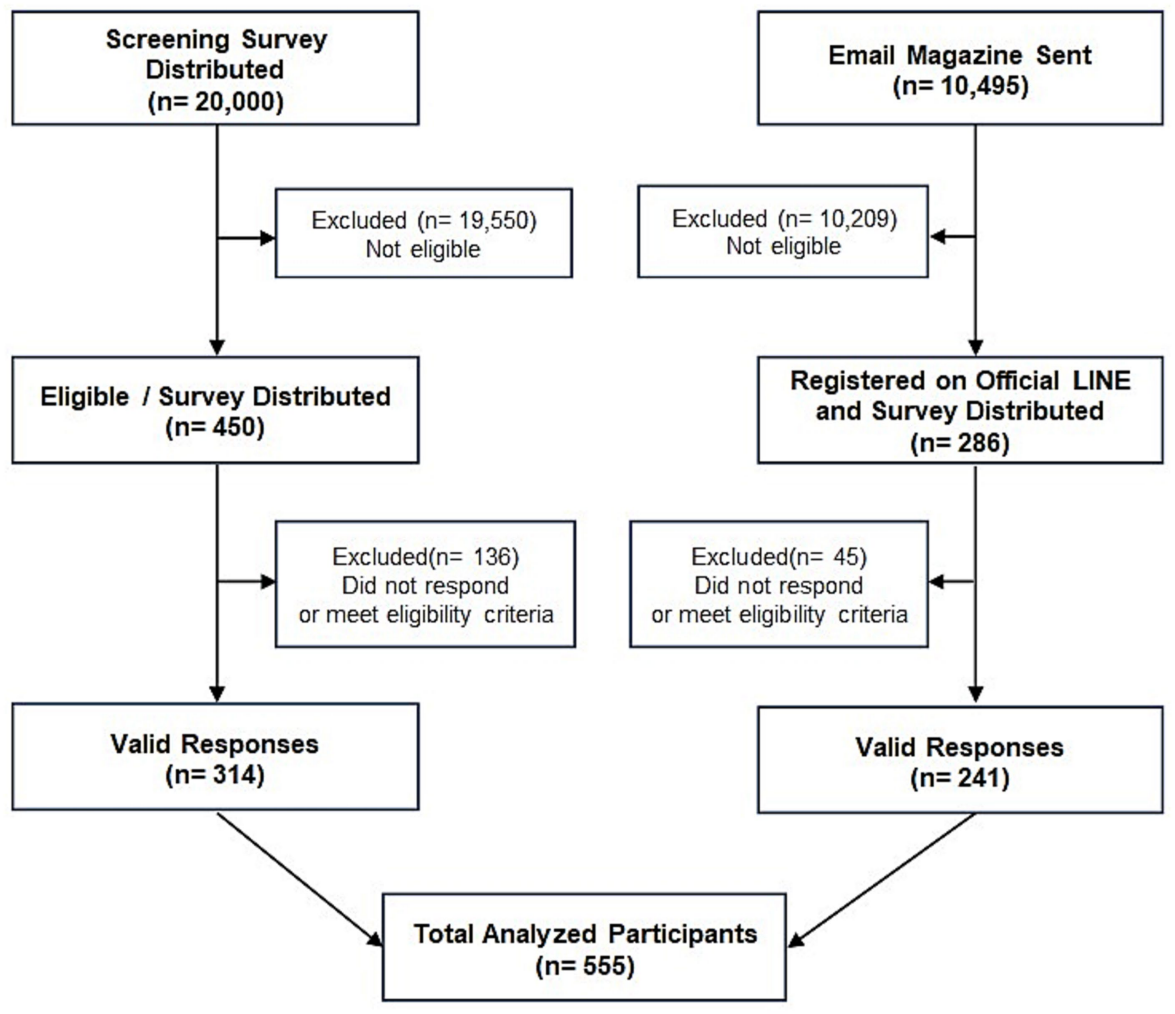
Flowchart of the study.

Figure 2 illustrates the participant recruitment flow, including the numbers of individuals screened, deemed eligible, invited to complete the survey, and included in the final analytic sample. Among participants who were eligible and received the survey, the valid completion rates were 69.8% (314/450) for the online research-company channel and 84.3% (241/286) for the email/LINE channel, yielding an overall valid completion rate of 75.4% (555/736).

Sample size was determined based on three criteria: (1) general recommendations for structural equation modeling (Kline, 2016), (2) anticipated response rates informed by prior studies, and (3) the ratio of sample size to the number of estimated parameters (Bentler and Chou, 1987). As a pragmatic rule-of-thumb, we aimed for at least 10 cases per observed variable. Because the final model included 16 observed variables, a minimum of 160 participants was required for a single-group model. Given the planned two-group SEM (primiparous vs. multiparous), this criterion was applied to each group, yielding a minimum total sample size of 320 participants. The final analytic sample (*N* = 555; primiparous *n* = 330; multiparous *n* = 225) exceeded this minimum requirement.

### 2.2 Measures

#### 2.2.1 Fathers’ paternity leave uptake and duration

Mothers reported whether the father had taken paternity leave and its duration. Accordingly, participants were categorized into three groups: (1) no, (2) short (<1 month), and (3) long leave (≥1 month). The one-month threshold was selected based on both the Japanese policy context and analytic considerations. In Japan, Childcare Leave at Birth, often referred to as postnatal paternity leave, allows eligible workers to take up to 4 weeks, or 28 days, within the first 8 weeks after childbirth, separately from standard childcare leave. Therefore, leave of <1 month approximates leave taken within this early postnatal-leave framework, whereas leave of ≥1 month captures longer leave extending beyond this initial period. The threshold also provided sufficient cell sizes for parity-stratified SEM. All measures were collected at 3 months postpartum.

#### 2.2.2 Paternal involvement in childcare and housework

The Child Care Activities and Housework Scale (Hinokuma et al., 1999) was used to assess paternal involvement in childcare and housework. The scale comprises 19 items organized into four domains: Interactional Activities (5 items), Concrete Childcare Activities (6 items), Mental Support (4 items), and Housework (4 items). Items are rated on a 4-point scale, with higher scores indicating more frequent involvement. Subsequent research has also scored and reported these four domains separately and has documented adequate internal consistency (Takimoto et al., 2020). Mean scores were calculated for each domain; Cronbach’s *α* coefficients in the present sample were 0.749, 0.843, 0.800, and 0.770, respectively. In the SEM, the four domain scores were retained separately because identifying domain-specific associations was a central study aim. Published evidence for formal construct validity beyond internal consistency remains limited, which should be considered when interpreting the findings.

#### 2.2.3 Coparenting relationship

The Japanese version (Takeishi et al., 2017) of the Coparenting Relationship Scale (CRS; Feinberg et al., 2012) was used to assess mothers’ perceived coparenting quality. The CRS comprises 35 items across seven subscales: Agreement, Closeness, Exposure to Conflict, Support, Undermining, Endorsement of Partner’s Parenting, and Division of Labor. The original study evaluated both subscale scores and the 35-item overall measure and documented internal consistency, temporal stability, construct validity, and interparent agreement (Feinberg et al., 2012). Reliability and validity of the Japanese version have also been examined (Takeishi et al., 2017; Nakamura et al., 2021). Because coparenting was prespecified as a global dyadic relational construct in the present model, we used the overall CRS score rather than the individual subscale scores. Items were scored so that higher overall scores indicated better coparenting quality. In our sample, Cronbach’s *α* was 0.845.

#### 2.2.4 Parenting resilience and adaptation

Maternal parenting resilience and adaptation were assessed using the Comprehensive Scale for Parenting Resilience and Adaptation (CPRA; Sugao et al., 2022). The CPRA comprises 81 items and 21 factors organized into five domains: Child’s Temperament and Health (D1), Environmental Resources (D2), Perceived Support (D3), Mother’s Cognitive and Behavioral Characteristics (D4), and Psychological Adaptation to Parenting (D5). Items were rated on a 5-point scale ranging from 1 (“strongly disagree”) to 5 (“strongly agree”). The original development study identified this five-domain organization and reported internal consistency for the constituent factors, as well as associations between all CPRA factors and depressive symptoms. Following scoring guidance provided by the scale developers, each domain score was calculated as the mean of the items assigned to that domain. Domain-level CPRA scores have also been used in a subsequent empirical study (Shima et al., 2024). Higher scores indicate greater parenting-related difficulties or poorer psychological adaptation. Based on the original domain definitions, D2–D4 represent distinct environmental, support-related, and maternal characteristic domains, whereas D5 represents Psychological Adaptation to Parenting (Sugao et al., 2022). In the present SEM, these original domains were retained and assigned prespecified analytic roles: D2–D4 were treated as distinct psychosocial resource- or difficulty-related domains, whereas D5 was positioned as the parenting-adaptation outcome domain. These analytic roles did not modify the original CPRA scoring or domain structure. In our sample, Cronbach’s *α* coefficients were 0.654 (D1), 0.783 (D2), 0.791 (D3), 0.835 (D4), and 0.889 (D5). CPRA D1 was excluded from the SEM because it showed relatively low internal consistency and limited variability in the current sample. This decision was made after examining reliability and distributional statistics and before SEM estimation. D1 primarily reflects infant temperament and health rather than the maternal psychosocial resources and parenting adaptation emphasized in the theory-guided model.

#### 2.2.5 Depressive symptoms

Maternal depressive symptoms were measured using the Japanese version of the Center for Epidemiologic Studies Depression Scale (CES-D; Shima et al., 1985), originally developed by Radloff (1977). This 20-item self-reported questionnaire was developed to screen depressive symptoms in the general population. Items enquired about the frequency of depression-related symptoms during the past week. Responses were rated on a 4-point Likert scale ranging from 0 (rarely or none of the time, <1 day) to 3 (most or all of the time, 5–7 days). Four positive items were reverse-coded. Total scores ranged from 0–60 points, and higher scores indicated more severe depressive symptoms. We used the total CES-D score as a continuous variable. In our sample, Cronbach’s *α* was 0.873.

Table 1 presents the details of scales, subscales, number of items, and Cronbach’s α coefficients for this sample.

**Table 1:** Scales, subscales, number of items, and reliability (Cronbach’s α).

Scale	Subscale	α	Number of items
Child care activities and Housework	Interactional Activities	0.749	5
	Concrete Child Care Activities	0.843	6
	Mental Support	0.800	4
	Housework	0.770	4
CRS	Agreement	0.822	4
	Closeness	0.794	5
	Exposure to conflict	0.875	5
	Support	0.896	6
	Undermining	0.842	6
	Endorsement of partner’s parenting	0.838	7
	Division of labor	0.592	2
	CRS Total Score	0.845	35
CPRA	D1 Child’s Temperament and Health	0.654	5
	D2 Environmental Resources	0.783	20
	D3 Perceived Support	0.791	15
	D4 Mother’s Cognitive and Behavioural Characteristics	0.835	22
	D5 Psychological Adaptation to Parenting	0.889	19
CES-D	Depressive Symptoms Total Score	0.873	20

CRS, Coparenting Relationship Scale; CPRA, Comprehensive Scale for Parenting Resilience and Adaptation; CES-D = Center for Epidemiologic Studies Depression Scale.

### 2.3 Statistical analysis

Analyses were conducted using IBM SPSS Statistics version 30.0 and Amos version 30.0. The online survey was configured to require responses to the analytic items. Therefore, there were no missing values for variables included in the SEM, and analyses were conducted using the complete analytic sample of 555 mothers. First, descriptive statistics and Pearson’s correlation coefficients were calculated. One-way analysis of variance (ANOVA) and chi-square tests were conducted to examine group differences by paternity leave status (no, short, long). Effect sizes were interpreted using conventional benchmarks (Cohen, 1988). For η^2^ and partial η^2^, values of 0.01, 0.06, and 0.14 were considered small, medium, and large, respectively. For Cramer’s V, benchmarks were adjusted according to k = min (r − 1, c − 1): 0.10, 0.30, and 0.50 for k = 1, and 0.07, 0.21, and 0.35 for k = 2. These values were used as general guidelines rather than absolute cutoffs.

Second, multi-group SEM was performed to examine whether paternity leave duration was related to maternal depressive symptoms via paternal involvement, coparenting, and maternal adaptation. Indirect effects and confidence intervals were evaluated using 5,000 bootstrap resamples with bias-corrected percentile 95% confidence intervals (two-tailed). Path estimates were compared descriptively across parity groups using standardized coefficients and 95% bias-corrected bootstrap confidence intervals; we did not impose cross-group equality constraints to formally test between-group differences in individual paths. Short and long paternity leave were entered as two dummy variables, with the no-leave group serving as the reference category throughout all SEM analyses. Mothers’ and fathers’ ages, mothers’ employment status, and perceived financial leeway were included as covariates. Covariances were specified among all the exogenous variables.

Endogenous variables included the four paternal involvement subscales, CRS total score, CPRA domains D2–D5, and maternal depressive symptoms (the CES-D total score). Paths were specified from paternity leave and covariates to each paternal involvement subscale and the four paternal involvement subscales to the CRS total score. Paths were specified from the CRS total score to CPRA D2, D3, D4, and D5; from CPRA D2–D4 to D5; and from CPRA D2–D5 to the CES-D total score. These directions reflected the theory-informed analytic ordering described in the Introduction and were not interpreted as temporal or causal effects. As reported above, CPRA D1 was excluded.

All theoretically specified structural paths were retained in the SEM. Path support was evaluated using 95% bias-corrected bootstrap confidence intervals: paths whose confidence intervals did not include zero were interpreted as supported, whereas paths whose confidence intervals included zero were retained in the model but interpreted as not clearly supported. No theoretically specified structural paths were deleted; therefore, the number of removed structural paths was zero. After inspecting modification indices, we added eight residual covariance pairs in each parity group, resulting in 16 additional residual covariance parameters across the two groups. These residual covariances were specified among conceptually related subdomains measured by the same instrument, including the paternal involvement domains and selected CPRA-related domains, to account for shared measurement sources and domain overlap. These modifications were limited to measurement-level residual covariances; no post-hoc structural paths were added. Modification indices were inspected for diagnostic purposes; however, we did not add post-hoc parameters (e.g., correlated residuals) unless theoretically justified, to avoid overfitting. Standardized path coefficients (*β*) and 95% bias-corrected bootstrap CIs were reported. Because this was a cross-sectional study, SEM results only described associations among the variables and did not constitute strict causal inferences.

### 2.4 Ethical considerations

An online information sheet explained the study’s purpose and ethical considerations. Only participants who checked a box that indicated informed consent could access the questionnaire. This study was approved by the Ethics Committee of The University of Osaka Hospital (Approval No. 24576).

## 3 Results

### 3.1 Preliminary analysis

#### 3.1.1 Participants’ characteristics

The mean age of mothers (3 months postpartum) and fathers (*n* = 555 for both) was 32.96 (SD = 4.66) and 34.58 years (SD = 5.94), respectively. Of the sample, 252 (45.4%), 134 (24.1%), and 169 (30.5%) fathers took no, short (<1), and long (≥1 month) paternity leave, respectively. Within the short leave group (<1 month; *n* = 134), reported leave duration was <1 (*n* = 8, 6.0%), <2 (*n* = 32, 23.9%), <3 (*n* = 2, 1.5%), <4 weeks (*n* = 3, 2.2%), and <1 month (*n* = 89, 66.4%). There were 330 primiparous (59.6%) and 225 multiparous (40.4%) mothers, with 73.0% employed and 27.0% unemployed. Table 2 presents the participants’ background characteristics according to leave group.

**Table 2:** Comparison of demographic characteristics according to father’s paternity leave groups.

Variables (*N* = 555)	Total (*n* = 555)	No paternity leave (*n* = 252)	Short paternity leave <1 month^a^ (*n* = 134)	Long paternity leave ≥1 month (*n* = 169)	p (ANOVA/Welch/χ^2^)	η2/Cramer’s V
Maternal age	32.96 ± 4.66	33.15 ± 4.78	32.80 ± 4.58	32.82 ± 4.56	0.697	0.001
Paternal age	34.58 ± 5.94	35.07 ± 6.38	34.27 ± 5.24	34.08 ± 5.75	0.194	0.006
Parity					<0.001	0.206
Primiparous	330 (59.6)	122 (48.4)	94 (70.1)	114 (67.5)		
Multiparous	225 (40.4)	130 (51.6)	40 (29.9)	55 (32.5)		
Feeding method at 3 months					0.014	0.106
Breastfeeding	182 (32.8)	77 (30.6)	49 (36.6)	56 (33.1)		
Formula	126 (22.7)	74 (29.4)	20 (14.9)	32 (18.9)		
Breastfeeding and formula	247 (44.5)	101 (40.0)	65 (48.5)	81 (47.9)		
Mode of delivery					0.080	0.101
Vaginal delivery	334 (60.2)	165 (65.5)	79 (58.9)	90 (53.3)		
Epidural analgesia	119 (21.4)	40 (15.9)	32 (23.9)	47 (27.8)		
Planned cesarean section	53 (9.5)	24 (9.5)	10 (7.5)	19 (11.2)		
Emergency cesarean section	49 (8.8)	23 (9.1)	13 (9.7)	13 (7.7)		
Maternal employment status					0.001	0.154
Employed	405 (73.0)	165 (65.5)	107 (79.9)	133 (78.7)		
Unemployed	150 (27.0)	87 (34.5)	27 (20.1)	36 (21.3)		
Economic status					0.379	0.076
Very little financial leeway	55 (9.9)	18 (7.1)	17 (12.7)	20 (11.8)		
Limited financial leeway	144 (25.9)	72 (28.6)	31 (23.1)	41 (24.3)		
Moderate financial leeway	310 (55.9)	138 (54.8)	78 (58.2)	94 (55.6)		
Considerable financial leeway	46 (8.3)	24 (9.5)	8 (6.0)	14 (8.3)		

a Within the Short Leave group (*n* = 134), the breakdown of duration was: < 1 week (*n* = 8, 6.0%), 1–2 (*n* = 32, 23.9%), 2–3 (*n* = 2, 1.5%), 3–4 weeks (*n* = 3, 2.2%), and 4 weeks to < 1 month (*n* = 89, 66.4%).

*p*-values were calculated using *χ*^2^ tests and one-way ANOVA for categorical and continuous variables, respectively. ***p < 0.001.

#### 3.1.2 Comparisons of background characteristics and key variables across the three paternity leave groups

Table 2 shows group differences in background characteristics according to fathers’ paternity leave status. Background variables differed across leave groups for parity (*p* < 0.001, Cramer’s V = 0.206), maternal employment status (*p* = 0.001, Cramer’s V = 0.154), and feeding method at 3 months postpartum (*p* = 0.014, Cramer’s V = 0.106). Descriptively, the short- and long-leave groups contained higher proportions of primiparous mothers (70.1 and 67.5%, respectively) than the no-leave group (48.4%) and higher proportions of employed mothers (79.9 and 78.7% vs. 65.5%). Formula feeding was more common in the no-leave group (29.4%) than in the short- and long-leave groups (14.9 and 18.9%), whereas mixed feeding was more common in the two leave-taking groups (48.5 and 47.9%) than in the no-leave group (40.0%); breastfeeding proportions were relatively similar across groups (30.6–36.6%). These percentages are descriptive because pairwise post-hoc comparisons were not conducted. No significant group differences were observed for maternal age (*p* = 0.697, η^2^ = 0.001), paternal age (*p* = 0.194, η^2^ = 0.006), mode of delivery (*p* = 0.080, Cramer’s V = 0.101), or perceived economic status (*p* = 0.379, Cramer’s V = 0.076).

Table 3 shows group differences in the key study variables. Significant group differences were observed for all four domains of fathers’ childcare and housework involvement (all *p* < 0.001; partial η^2^ = 0.039–0.085), with the long-leave group generally showing the most favorable pattern. Coparenting quality also differed across leave groups, including the CRS total score (*p* < 0.001, partial η^2^ = 0.051). Regarding maternal resilience/adaptation, significant group differences were observed for Environmental Resources (CPRA D2; *p* < 0.001, partial η^2^ = 0.026), Perceived Support (CPRA D3; *p* = 0.005, partial η^2^ = 0.019), and Psychological Maladaptation to Parenting (CPRA D5; *p* = 0.002, partial η^2^ = 0.022). Maternal depressive symptoms also differed across groups, although the effect size was small (CES-D; *p* = 0.034, partial η^2^ = 0.012). No significant group differences were observed for CPRA D1 (*p* = 0.308, partial η^2^ = 0.004) or CPRA D4 (*p* = 0.147, partial η^2^ = 0.007).

**Table 3:** Comparison of the key study variables according to father’s paternity leave groups.

Variables (*N* = 555)	Total (*n* = 555)	No paternity leave (*n* = 252)	Short paternity leave <1 month^a^ (*n* = 134)	Long Paternity Leave ≥1 month (*n* = 169)	p (ANOVA)	η^2^ (partial)
Child care activities and housework						
Interactional activities	3.11 ± 0.57	2.98 ± 0.62	3.13 ± 0.48	3.29 ± 0.52	<0.001	0.056
Concrete child care activities	2.81 ± 0.72	2.60 ± 0.72	2.86 ± 0.65	3.08 ± 0.69	<0.001	0.081
Supporting mentally	2.95 ± 0.71	2.80 ± 0.74	3.03 ± 0.62	3.10 ± 0.69	<0.001	0.039
Housework	2.58 ± 0.78	2.35 ± 0.76	2.64 ± 0.68	2.88 ± 0.77	<0.001	0.085
Coparenting Relationship Scale (CRS)						
Agreement	4.37 ± 1.14	4.25 ± 1.22	4.65 ± 1.01	4.34 ± 1.09	0.004	0.020
Closeness	4.07 ± 1.21	3.82 ± 1.26	4.28 ± 1.07	4.29 ± 1.18	<0.001	0.037
Exposure to conflict	0.98 ± 1.02	1.04 ± 1.11	0.87 ± 0.94	0.98 ± 0.94	0.319	0.004
Support	3.46 ± 1.47	3.12 ± 1.51	3.74 ± 1.30	3.75 ± 1.42	<0.001	0.046
Undermining	1.17 ± 1.12	1.32 ± 1.17	0.99 ± 1.08	1.09 ± 1.05	0.015	0.015
Endorsement of partner’s parenting	3.62 ± 1.23	3.31 ± 1.25	3.83 ± 1.15	3.90 ± 1.18	<0.001	0.052
Division of labor	3.97 ± 1.56	3.62 ± 1.58	4.18 ± 1.44	4.32 ± 1.52	<0.001	0.043
CRS Total Score	2.97 ± 0.64	2.81 ± 0.67	3.08 ± 0.56	3.11 ± 0.61	<0.001	0.051
Comprehensive Scale for Parenting Resilience and Adaptation (CPRA)						
D1 Child’s Temperament and Health	2.14 ± 0.62	2.17 ± 0.64	2.07 ± 0.59	2.17 ± 0.62	0.308	0.004
D2 Environmental Resources	2.32 ± 0.52	2.40 ± 0.50	2.20 ± 0.48	2.30 ± 0.55	<0.001	0.026
D3 Perceived Support	2.26 ± 0.54	2.34 ± 0.52	2.21 ± 0.52	2.17 ± 0.57	0.005	0.019
D4 Mother’s Cognitive and Behavioural Characteristics	2.46 ± 0.51	2.49 ± 0.54	2.39 ± 0.47	2.46 ± 0.48	0.147	0.007
D5 Psychological Adaptation to Parenting	2.30 ± 0.60	2.37 ± 0.61	2.15 ± 0.51	2.32 ± 0.63	0.002	0.022
Depressive Symptoms						
CES-D Total Score	11.69 ± 9.10	12.67 ± 9.39	10.19 ± 9.00	11.41 ± 8.59	0.034	0.012

a Within the Short Leave group (*n* = 134), the breakdown of duration was: < 1 week (*n* = 8, 6.0%), 1–2 (*n* = 32, 23.9%), 2–3 (*n* = 2, 1.5%), 3–4 weeks (*n* = 3, 2.2%), and 4 weeks to < 1 month (*n* = 89, 66.4%).

CES-D, Center for Epidemiologic Studies Depression Scale; CRS, Coparenting Relationship Scale; CPRA, Comprehensive Scale for Parenting Resilience and Adaptation. Higher CPRA scores indicate greater difficulty.

SD, Standard Deviation; η^2^, partial eta-squared. ***p < 0.001, **p < 0.01.

#### 3.1.3 Correlations among the key variables

Table 4 presents the Pearson’s correlations among the key variables. All father-related variables reflected mothers’ reports of fathers’ behaviors and the coparenting relationship. Mothers’ ratings of fathers’ involvement domains showed moderate-to-strong intercorrelations and were consistently associated with better coparenting quality (higher CRS total and higher positive CRS subscales; lower negative CRS subscales). The CRS total score was negatively correlated with the CPRA D2 and D3 and negatively correlated with D5, indicating that better coparenting co-occurred with fewer perceived difficulties and lower maladaptation. The CES-D score had the strongest positive association with D5 and was also positively associated with D2 and D4. However, its association with the CRS was weaker but significant. Overall, the correlation pattern was consistent with the proposed serial associations assessed in the SEM.

**Table 4:** Correlation matrix of the study variables.

Variables	1	2	3	4	5	6	7	8	9	10	11	12	13	14	15	16	17	18
1. Interactional activities	1																	
2. Concrete child care activities	0.638^**^	1																
3. Mental support	0.590^**^	0.620^**^	1															
4. Housework	0.456^**^	0.556^**^	0.525^**^	1														
5. Agreement	0.275^**^	0.236^**^	0.386^**^	0.252^**^	1													
6. Closeness	0.420^**^	0.385^**^	0.506^**^	0.307^**^	0.591^**^	1												
7. Exposure to conflict	−0.205^**^	−0.172^**^	−0.187^**^	−0.085^*^	−0.439^**^	−0.382^**^	1											
8. Support	0.398^**^	0.413^**^	0.638^**^	0.357^**^	0.514^**^	0.758^**^	−0.282^**^	1										
9. Undermining	−0.278^**^	−0.234^**^	−0.294^**^	−0.218^**^	−0.703^**^	−0.513^**^	0.522^**^	−0.350^**^	1									
10. Endorsement of partner’s parenting	0.463^**^	0.470^**^	0.547^**^	0.380^**^	0.531^**^	0.738^**^	−0.297^**^	0.752^**^	−0.383^**^	1								
11. Division of labor	0.348^**^	0.425^**^	0.409^**^	0.407^**^	0.481^**^	0.450^**^	−0.277^**^	0.451^**^	−0.521^**^	0.582^**^	1							
12. Coparenting	0.422^**^	0.444^**^	0.602^**^	0.392^**^	0.525^**^	0.793^**^	−0.072	0.883^**^	−0.221^**^	0.885^**^	0.540^**^	1						
13. CPRA D1	−0.097^*^	−0.064	−0.086^*^	−0.087^*^	−0.285^**^	−0.253^**^	0.257^**^	−0.148^**^	0.348^**^	−0.145^**^	−0.176^**^	−0.102^*^	1					
14. CPRA D2	−0.318^**^	−0.284^**^	−0.390^**^	−0.298^**^	−0.562^**^	−0.597^**^	0.423^**^	−0.547^**^	0.503^**^	−0.579^**^	−0.423^**^	−0.524^**^	0.357^**^	1				
15. CPRA D3	−0.203^**^	−0.240^**^	−0.230^**^	−0.240^**^	−0.271^**^	−0.357^**^	0.154^**^	−0.288^**^	0.261^**^	−0.359^**^	−0.294^**^	−0.330^**^	0.128^**^	0.347^**^	1			
16. CPRA D4	−0.096^*^	−0.074	−0.089^*^	−0.050	−0.405^**^	−0.304^**^	0.309^**^	−0.163^**^	0.466^**^	−0.172^**^	−0.213^**^	−0.114^**^	0.421^**^	0.426^**^	0.177^**^	1		
17. CPRA D5	−0.176^**^	−0.139^**^	−0.232^**^	−0.139^**^	−0.452^**^	−0.472^**^	0.260^**^	−0.366^**^	0.454^**^	−0.334^**^	−0.244^**^	−0.331^**^	0.486^**^	0.497^**^	0.243^**^	0.673^**^	1	
18. Depressive symptoms (CES-D)	−0.191^**^	−0.151^**^	−0.264^**^	−0.150^**^	−0.332^**^	−0.343^**^	0.340^**^	−0.240^**^	0.415^**^	−0.243^**^	−0.235^**^	−0.179^**^	0.339^**^	0.417^**^	0.168^**^	0.517^**^	0.586^**^	1

CRS, Coparenting Relationship Scale. Variables 5–11 are CRS subscale scores (Agreement, Closeness, Exposure to conflict, Support, Undermining, Endorsement of partner’s parenting, and Division of labor). Variable 12 (Coparenting) is the overall CRS score (mean total score).

CPRA dimensions are coded as follows: D1 = Child’s Temperament and Health; D2 = Environmental Resources; D3 = Perceived Support; D4 = Mother’s Cognitive and Behavioural Characteristics; D5 = Psychological Adaptation to Parenting.

*p < 0.05, **p < 0.01.

### 3.2 Multi-group SEM

#### 3.2.1 Model specification and fit

Multi-group SEM was estimated for primiparous (*n* = 330) and multiparous (*n* = 225) mothers. The initial theory-specified model retained all hypothesized structural paths and covariances among exogenous variables, but did not include residual covariances among endogenous residuals. This initial model showed poor fit: *χ*^2^(84) = 1098.181, *p* < 0.001, *χ*^2^/df = 13.074, CFI = 0.644, TLI = −0.016, RMSEA = 0.148, 90% CI [0.140, 0.156], PCLOSE < 0.001.

After adding theoretically interpretable residual covariances among same-instrument subdomains, the final refined model showed acceptable fit: *χ*^2^(68) = 133.216, *p* < 0.001, *χ*^2^/df = 1.959, CFI = 0.977, TLI = 0.919, RMSEA = 0.042, 90% CI [0.031, 0.052], PCLOSE = 0.904. Fit indices for the initial and final refined models are presented in Table A1.

**Figure 3:**
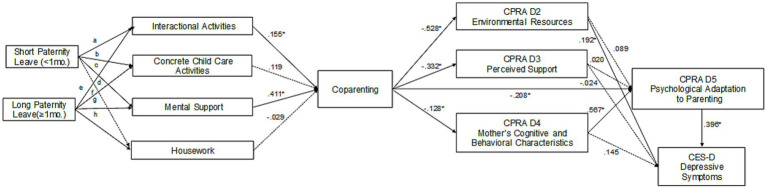
Final refined SEM model for primiparous mothers (*n* = 330). Standardized path coefficients (*β*) are shown. Solid and dashed lines indicate paths whose 95% bias-corrected percentile bootstrap confidence intervals (5,000 resamples; two-tailed) excluded and included zero, respectively. Paths from covariates (maternal age, paternal age, perceived financial leeway, and maternal employment status) were included in the model but are omitted for clarity. To reduce visual clutter, paths from paternity leave to paternal involvement are labeled a–h: Short leave (<1 month) to Interactional Activities (a = 0.186), to Concrete Childcare Activities (b = 0.198), to Mental Support (c = 0.161), to Housework (d = 0.109); Long leave (≥1 month) to Interactional Activities (e = 0.339), to Concrete Childcare Activities (*f* = 0.386), to Mental Support (g = 0.273), to Housework (h = 0.338). Model fit indices (unconstrained multi-group model): *χ*^2^(68) = 133.216, *p* < 0.001, *χ*^2^/df = 1.96, CFI = 0.977, TLI = 0.919, RMSEA = 0.042 [90% CI (0.031, 0.052), PCLOSE = 0.904].

**Figure 4:**
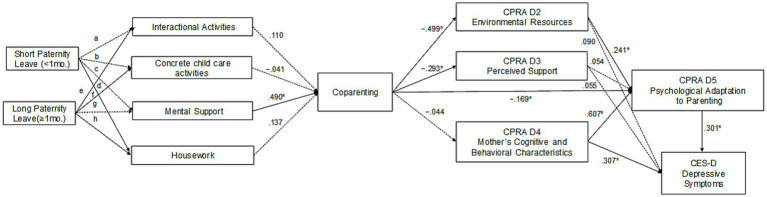
Final refined SEM model for multiparous mothers (*n* = 225). Standardized path coefficients (*β*) are shown. Solid and dashed lines indicate paths whose 95% bias-corrected percentile bootstrap confidence intervals (5,000 resamples; two-tailed) excluded and included zero, respectively. Paths from covariates (maternal age, paternal age, perceived financial leeway, and maternal employment status) were included in the model but are omitted for clarity. To reduce visual clutter, paths from paternity leave to paternal involvement are labeled a–h: Short leave (<1 month) to Interactional Activities (a = 0.104), to Concrete Childcare Activities (b = 0.088), to Mental Support (c = 0.107), to Housework (d = 0.188); Long leave (≥1 month) to Interactional Activities (e = 0.176), to Concrete Childcare Activities (*f* = 0.163), to Mental Support (g = 0.067), to Housework (h = 0.212). Model fit indices (unconstrained multi-group model): *χ*^2^(68) = 133.216, *p* < 0.001, *χ*^2^/df = 1.96, CFI = 0.977, TLI = 0.919, RMSEA = 0.042 [90% CI (0.031, 0.052), PCLOSE = 0.904].

Figures 3, 4 illustrate the final refined primary models for the primiparous and multiparous groups, respectively.

#### 3.2.2 Main paths

All coefficients for short and long paternity leave indicate associations relative to the no-leave reference group. In the primiparous group, compared with no leave, short paternity leave (<1 month) was positively associated with fathers’ Interactional [*β* = 0.186, 95% bias-corrected bootstrap CI (0.073, 0.287)] and Concrete Childcare Activities [*β* = 0.198, 95% CI (0.079, 0.314)] as well as Mental Support [*β* = 0.161, 95% CI (0.044, 0.279)]; however, the association with Housework was not supported [*β* = 0.109, 95% CI (−0.005, 0.224)]. Furthermore, long paternity leave (≥1 month) was positively associated with all four paternal involvement subscales (*β* = 0.273–0.386, all 95% CIs excluding zero). In the multiparous group, short leave was positively associated with Housework [*β* = 0.188, 95% CI (0.071, 0.300)], whereas associations with Interactional and Concrete Childcare Activities were not supported (bootstrap CIs included zero). Furthermore, long leave was positively associated with Interactional [*β* = 0.176, 95% CI (0.039, 0.304)] and Concrete Childcare Activities [*β* = 0.163, 95% CI (0.018, 0.306)] and Housework [*β* = 0.212, 95% CI (0.068, 0.337)] in the multiparous group. However, associations of both leave durations with Mental Support were not supported (bootstrap CIs included zero).

Fathers’ Mental Support had the strongest positive association with coparenting across both parity groups [primipara: *β* = 0.411, 95% CI (0.285, 0.527); multipara: *β* = 0.490, 95% CI (0.350, 0.612)]. In primiparous families, Interactional Activities were positively associated with coparenting (*β* = 0.155, 95% CI [0.027, 0.280]), whereas associations with Concrete Childcare Activities and Housework were not supported. In multiparous families, associations of Interactional and Concrete Childcare Activities and Housework with coparenting were not supported (all bootstrap CIs included zero). Regarding covariates, paternal age was negatively associated with coparenting among multiparous families [*β* = −0.144, 95% CI (−0.280, −0.005]).

In both groups, higher coparenting was associated with lower CPRA Environmental Resources [primipara: *β* = −0.528, 95% CI (−0.598, −0.442); multipara: *β* = −0.499, 95% CI (−0.592, −0.393)] and Perceived Support [primipara: *β* = −0.332, 95% CI (−0.419, −0.243); multipara: *β* = −0.293, 95% CI (−0.406, −0.173)]. Furthermore, coparenting was directly associated with lower Psychological Maladaptation [primipara: *β* = −0.208, 95% CI (−0.304, −0.115); multipara: *β* = −0.169, 95% CI (−0.269, −0.071)]. Psychological Maladaptation also had a positive association with depressive symptoms [primipara: *β* = 0.396, 95% CI (0.178, 0.571); multipara: *β* = 0.301, 95% CI (0.133, 0.459)]. In addition, Mother’s Cognitive/Behavioral Characteristics were positively associated with Psychological Maladaptation in both groups [primipara: *β* = 0.567, 95% CI (0.467, 0.657); multipara: *β* = 0.607, 95% CI (0.515, 0.690)]. A direct association was observed between Mother’s Cognitive/Behavioral Characteristics and depressive symptoms among multiparous families [*β* = 0.307, 95% CI (0.162, 0.444)]; however, this association was not observed in primiparous families [*β* = 0.145, 95% CI (−0.016, 0.311)]. Environmental Resources were positively associated with depressive symptoms among primiparous families [*β* = 0.192, 95% CI (0.097, 0.282)]; however, this association was also not observed in multiparous families [*β* = 0.090, 95% CI (−0.048, 0.218)]. Key standardized path coefficients, 95% confidence intervals, and support status for the final refined model are summarized in Table 5.

**Table 5:** Summary of supported and not clearly supported standardized structural paths in the final refined multi-group SEM.

Path	Primipara *β* [95% CI]	Primipara support status	Multipara β [95% CI]	Multipara support status
Panel A: paternity leave → paternal involvement				
Short leave → Interactional activities	0.186 [0.073, 0.287]	Supported	0.104 [−0.020, 0.213]	Not clearly supported
Long leave → Interactional activities	0.339 [0.225, 0.446]	Supported	0.176 [0.039, 0.304]	Supported
Short leave → Concrete childcare activities	0.198 [0.079, 0.314]	Supported	0.088 [−0.027, 0.206]	Not clearly supported
Long leave → Concrete childcare activities	0.386 [0.277, 0.485]	Supported	0.163 [0.018, 0.306]	Supported
Short leave → Housework activities	0.109 [−0.005, 0.224]	Not clearly supported	0.188 [0.071, 0.300]	Supported
Long leave → Housework activities	0.338 [0.219, 0.449]	Supported	0.212 [0.068, 0.337]	Supported
Short leave → Mental support behavior	0.161 [0.044, 0.279]	Supported	0.107 [−0.020, 0.223]	Not clearly supported
Long leave → Mental support behavior	0.273 [0.153, 0.387]	Supported	0.067 [−0.081, 0.202]	Not clearly supported
Panel B: paternal involvement → coparenting (CRS)				
Interactional activities → Coparenting	0.155 [0.027, 0.280]	Supported	0.110 [−0.125, 0.327]	Not clearly supported
Concrete childcare activities → Coparenting	0.119 [−0.005, 0.251]	Not clearly supported	−0.041 [−0.200, 0.103]	Not clearly supported
Housework activities → Coparenting	−0.029 [−0.139, 0.079]	Not clearly supported	0.137 [−0.007, 0.277]	Not clearly supported
Mental support behavior → Coparenting	0.411 [0.285, 0.527]	Supported	0.490 [0.350, 0.612]	Supported
Panel C: downstream paths to depressive symptoms (CES-D)				
Coparenting → Environmental Resources (CPRA D2)	−0.528 [−0.598, −0.442]	Supported	−0.499 [−0.592, −0.393]	Supported
Coparenting → Perceived Support (CPRA D3)	−0.332 [−0.419, −0.243]	Supported	−0.293 [−0.406, −0.173]	Supported
Coparenting → Mother’s Cognitive/Behavioral Characteristics (CPRA D4)	−0.128 [−0.237, −0.023]	Supported	−0.044 [−0.177, 0.083]	Not clearly supported
Coparenting → Psychological Maladaptation (CPRA D5)	−0.208 [−0.304, −0.115]	Supported	−0.169 [−0.269, −0.071]	Supported
Mother’s Cognitive/Behavioral Characteristics (CPRA D4) → Psychological Maladaptation (CPRA D5)	0.567 [0.467, 0.657]	Supported	0.607 [0.515, 0.690]	Supported
Environmental Resources (CPRA D2) → Psychological Maladaptation (CPRA D5)	0.089 [−0.026, 0.191]	Not clearly supported	0.241 [0.121, 0.358]	Supported
Perceived Support (CPRA D3) → Psychological Maladaptation (CPRA D5)	0.020 [−0.064, 0.109]	Not clearly supported	0.054 [−0.033, 0.140]	Not clearly supported
Psychological Maladaptation (CPRA D5) → Depressive symptoms (CES-D)	0.396 [0.178, 0.571]	Supported	0.301 [0.133, 0.459]	Supported
(Direct) Environmental Resources (CPRA D2) → Depressive symptoms (CES-D)	0.192 [0.097, 0.282]	Supported	0.090 [−0.048, 0.218]	Not clearly supported
(Direct) Perceived Support (CPRA D3) → Depressive symptoms (CES-D)	−0.024 [−0.111, 0.068]	Not clearly supported	0.055 [−0.055, 0.165]	Not clearly supported
(Direct) Mother’s Cognitive/Behavioral Characteristics (CPRA D4) → Depressive symptoms (CES-D)	0.145 [−0.016, 0.311]	Not clearly supported	0.307 [0.162, 0.444]	Supported

Values are standardized coefficients (β) with 95% bias-corrected bootstrap confidence intervals based on 5,000 resamples from the final refined multi-group SEM (primipara *n* = 330; multipara *n* = 225). Paths were classified as “Supported” when the 95% confidence interval did not include zero and as “Not clearly supported” when the 95% confidence interval included zero. Short and long paternity leave were dummy-coded, with the no-leave group as the reference category. Paternal involvement was modeled using four observed subscales: Interactional Activities, Concrete Childcare Activities, Housework Activities, and Mental Support Behavior. Covariate paths and residual covariances were estimated but are omitted from this table for brevity. SEM, structural equation modeling; CRS, Coparenting Relationship Scale; CPRA, Comprehensive Scale for Parenting Resilience and Adaptation; CES-D, Center for Epidemiologic Studies Depression Scale.

#### 3.2.3 Total indirect associations of paternity leave with CPRA D5 and the CES-D

Total indirect associations with CPRA D5 were negative and supported for both short leave (β_total = −0.038, 95% CI [−0.071, −0.016]) and long leave [β_total = −0.067, 95% CI (−0.104, −0.042)] in the primiparous group. In the multiparous group, the association was supported for short leave [β_total = −0.029, 95% CI (−0.059, −0.002)] but was not clearly supported for long leave [β_total = −0.025, 95% CI (−0.062, 0.008)]. Since no direct paths from paternity leave to the CES-D were specified, total effects represented indirect effects through the modeled pathways. In the primiparous group, both short and long leave had significant negative total indirect associations on the CES-D [Short: β_total = −0.028, 95% CI (−0.054, −0.012); Long: β_total = −0.049, 95% CI (−0.077, −0.029)]. Furthermore, short leave had a significant negative total indirect effect on the CES-D in the multiparous group [β_total = −0.015, 95% CI (−0.035, −0.001)], whereas the total indirect association of long leave was not clearly supported [β_total = −0.013, 95% CI (−0.037, 0.002)]. These bootstrapped standardized total indirect effects are presented in Table 6.

**Table 6:** Bootstrapped standardized total indirect associations with maternal adaptation (CPRA D5) and depressive symptoms (CES-D).

Outcome	Effect	Primipara (*n* = 330)	Multipara (*n* = 225)
Maternal adaptation	Short leave	**−0.038 [−0.071, −0.016]**	**−0.029 [−0.059, −0.002]**
Maternal adaptation	Long leave	**−0.067 [−0.104, −0.042]**	−0.025 [−0.062, 0.008]
Depressive symptoms	Short leave	**−0.028 [−0.054, −0.012]**	**−0.015 [−0.035, −0.001]**
Depressive symptoms	Long leave	**−0.049 [−0.077, −0.029]**	−0.013 [−0.037, 0.002]

Data are standardized estimates with 95% bias-corrected percentile bootstrap confidence intervals based on 5,000 resamples (two-tailed). Short and long paternity leave are dummy variables (reference = no leave). Models were adjusted for mothers’ age, fathers’ age, perceived financial leeway, and mothers’ employment status. Bold values indicate statistical significance (95% CI does not include zero).

### 3.3 Verification of the hypotheses

The association between paternity leave and paternal involvement was clearer in primiparous families, especially for long leave, whereas in multiparous families leave was mainly associated with housework and selected task-based domains. Moreover, not all paternal involvement domains were associated with coparenting quality; across both groups, mental support showed the clearest association with coparenting quality. Therefore, H1 was partially supported, and the pattern was more clearly observed among primiparous families.

Higher coparenting quality was associated with fewer perceived difficulties in key parenting resilience/adaptation domains and lower psychological maladaptation to parenting, and psychological maladaptation was associated with depressive symptoms in both parity groups. Therefore, H2 was generally supported as a pattern of cross-sectional associations. Because the study was cross-sectional, these findings should be interpreted as theory-guided associations rather than evidence of temporal or causal processes.

## 4 Discussion

We examined associations of paternity leave group (no leave, short <1 month, or long ≥1 month) with maternal parenting adaptation and depressive symptoms at 3 months postpartum using parity-stratified multi-group SEM. The final model showed a theory-guided pattern of indirect associations among paternity leave group, mothers’ reports of fathers’ involvement, mothers’ perceived coparenting quality, CPRA domains, and depressive symptoms. Because all measures of paternal involvement and coparenting were based on mothers’ reports, references to paternal involvement and coparenting in the Discussion denote mothers’ reports or perceptions unless otherwise specified. We discuss (1) group-specific patterns linking leave group with fathers’ involvement and perceived coparenting, (2) perceived coparenting as a relational correlate of maternal parenting resources and adaptation, and (3) indirect associations with depressive symptoms, followed by policy and perinatal practice implications. Because all variables were measured cross-sectionally, the ordering of these associations should not be interpreted as temporal or causal.

A key implication of our findings is that the potential relevance of paternity leave for maternal postpartum adjustment may depend not only on whether fathers take leave and for how long, but also on the extent and pattern of their childcare, housework, and supportive involvement at 3 months postpartum, as reported by mothers. In particular, mothers’ reports of fathers’ Mental Support showed the strongest association with mothers’ perceived coparenting quality across parity groups.

### 4.1 Associations of leave duration with fathers’ involvement domains and coparenting

A key insight from the multi-group SEM was that fathers’ involvement was multidimensional in its associations with both paternity leave and coparenting quality. We focused on group-specific patterns of the observed associations in primiparous and multiparous families. Although some paths were statistically supported in only one parity group, these analyses primarily described group-specific associations rather than definitive evidence that individual coefficients differed between groups. Policy evaluations suggest that leave design—particularly reserving non-transferable time for fathers—can shape whether leave uptake translates into increased paternal engagement in unpaid work and care (Patnaik, 2019). Similarly, observational research linked fathers’ leave-taking to greater subsequent involvement in childcare and housework and multiple dimensions of father engagement (Bünning, 2015; Knoester et al., 2019; Petts and Knoester, 2018).

Both short and long leave were associated with higher involvement in Interactional and Concrete Childcare Activities in primiparous families; furthermore, they were linked to higher Mental Support. Additionally, long leave had a clear association with Housework. This pattern is compatible with the interpretation that for first-time parents, leave may coincide with opportunities for increased caregiving time and developing shared understanding, planning, and emotional/psychological support within the couple—elements that may be salient during the transition to parenthood.

Notably, the short leave category (<1 month) dominated the leave durations close to 1 month (approximately 1 month: 66.4%), whereas very brief leave (≤2 weeks) accounted for 29.9% of the short-leave group. This distribution suggests that our short-leave group largely reflects near-month leave rather than only very brief leave. This may help us partly interpret why short leave was associated with multiple involvement domains, especially in primiparous families.

In contrast, among multiparous families, leave was associated primarily with Housework (short leave) and Housework along with modest increases in Interactional and Concrete Childcare Activities (long leave). However, Mental Support was not clearly associated with any leave. This pattern may reflect the different constraints of multiparous households (e.g., established routines, older-child care needs, time scarcity); leave time may be absorbed by logistical and task-oriented demands rather than translating into increased psychological support or relational coordination.

Furthermore, our model clarifies the involvement domains uniquely associated with coparenting quality, accounting for correlations among the involvement subscales. Mental Support had the largest independent positive association with the total CRS score in both parity groups. Nevertheless, Mental Support shares supportive content with the CRS Support domain, and both measures were based on mothers’ reports. Conceptual overlap and common-method variance may therefore have contributed to the magnitude of this association; accordingly, the two constructs should not be regarded as entirely independent in the present study. In addition, Interactional Activities had a small, supported association with the total CRS score in primiparas; the coefficient for Housework in multiparas was positive but not clearly supported (the bootstrap CI included zero). In contrast, Concrete Childcare Activities had no clear unique association with the total CRS score after other involvement domains were included. These findings suggest that coparenting quality may be related less to “doing more childcare” and more to how fathers psychologically support mothers and coordinate parenting as a team (e.g., anticipating needs, sharing mental load, validating, and collaboratively solving problems; Daminger, 2019). This interpretation aligns with Feinberg’s theory positioning coparenting as a distinct family subsystem centered on coordination, mutual support, and shared decision-making (2003). These results also align with those of previous research that day-to-day couple closeness and coparenting support mutually influenced each other during the transition to parenthood (Le et al., 2019).

At the same time, contextual and selection processes constrain the interpretation of these associations. The observed patterns should not be interpreted as showing that leave duration itself produced more supportive paternal involvement. In Japan, policy design and actual leave duration may shape whether leave affords sustained opportunities for caregiving or is experienced as a brief and stressful transition. Access to leave and the capacity to translate leave time into supportive involvement may also depend on job conditions, workplace norms and support, anticipated career disadvantages, concern about burdening colleagues, and pre-existing family resources (Patnaik, 2019; Pizarro and Gartzia, 2024; Schaber et al., 2021). Self-selection may operate in either direction: supportive workplaces and stronger pre-existing family resources may facilitate both leave-taking and favorable family functioning, whereas greater caregiving demands or pre-existing family difficulties may prompt fathers to take leave. Fathers’ caregiving preparation, confidence, social support, and psychological adjustment may further shape how leave is experienced and how they engage with mothers and infants. The adverse association between paternity leave and father–infant bonding reported in Japan may therefore partly reflect contextual stressors, fathers’ psychological adjustment, or selection into leave rather than a harmful effect of leave itself (Terada et al., 2022). Because the present study did not measure workplace support, reasons for leave-taking, caregiving preparation, or paternal mental health, these explanations could not be tested. Future longitudinal studies should assess these factors, together with leave timing and duration, to distinguish leave-related associations from contextual and selection processes.

### 4.2 Coparenting quality as a relational context associated with maternal resilience and adaptation

Across both parity groups, coparenting quality had robust cross-sectional associations with multiple maternal resilience/adaptation domains. Higher CRS was consistently associated with fewer perceived difficulties in Environmental Resources (D2) and Perceived Support (D3) and lower Psychological Maladaptation to Parenting (D5). These associations are consistent with conceptualizations of coparenting as a relational resource that co-occurs with mothers perceiving greater support and fewer constraints. Furthermore, these results are linked to more favorable early postpartum adjustment. They also align with findings of previous studies that higher-quality coparenting was associated with lower maternal depressive symptoms and more adaptive parent–infant bonding (Estlein and Shai, 2024; Takeishi et al., 2021). Furthermore, CRS has been increasingly applied cross-culturally, including in a recent validation study with parents of infants and young children in Spain (Seijo et al., 2024).

Our model further indicated that mothers’ Cognitive/Behavioral Characteristics difficulties (D4) were strongly associated with D5 in both groups. Thus, these results highlight the relevance of individual vulnerability and coping tendencies. However, coparenting retained an independent association with D5, even after accounting for D4. This pattern may be reflective of the idea that supportive coparenting may be related to how manageable mothers perceive challenges, their engagement in problem-solving, and emotional security during early postpartum adjustment (Adlington et al., 2023).

Notably, the model also showed group-specific patterns among resilience/adaptation domains: D2 had an additional positive association with D5 in multiparas; however, no clear association was observed in primiparas. This may suggest that for multiparous families, environmental constraints (e.g., time, childcare availability, competing demands) are more closely tied to maladaptive adjustment difficulties, which may be explained by higher and more chronic demands.

### 4.3 Group-specific patterns in associations with depressive symptoms and indirect pathways

Consistent with prior studies conceptualizing postpartum depressive symptoms as emerging from interactions between vulnerability and contextual stressors (Biaggi et al., 2016; Norhayati et al., 2015), our model revealed that D5 was robustly and consistently associated with the CES-D scores in both parity groups. Furthermore, additional associations showed different descriptive patterns across the two parity groups.

Among primiparas, D2 had a small direct association with depressive symptoms, suggesting that perceived resource constraints may be related to depressive symptoms beyond their association through broader maladaptation. Maternal age also had a small protective association with the CES-D. D4 demonstrated a small positive correlation with the CES-D scores in primiparas; however, since the bootstrap confidence interval crossed zero, the robustness of this association is uncertain and should be interpreted cautiously.

Among multiparas, a different group-specific pattern was observed: D4 had a robust positive association with the CES-D, whereas D2 had no clear direct association. In addition, the financial indicator (Financial) had a small positive association with the CES-D, consistent with the possibility that greater financial constraints/strain are associated with higher depressive symptoms in multiparous mothers. These results suggest that depressive symptoms may be more tightly associated with individual D4 and structural/economic pressures in multiparas. Meanwhile, D2 may operate more through their association with psychological maladaptation (D2–D5) rather than directly with the CES-D scores. Because formal equality constraint tests were not conducted, these parity-related findings should be interpreted as group-specific patterns rather than statistically confirmed differences between primiparous and multiparous mothers.

These group-specific patterns help contextualize the total indirect associations between paternity leave and maternal depressive symptoms. Notably, depressive symptoms differed significantly across leave groups in the preliminary (unadjusted) comparisons, although the effect size was small (Table 3). Thus, any leave-related differences were likely small. In primiparous families, both leaves had negative total (indirect) associations with depressive symptoms, consistent with an indirect pathway in which leave-taking co-occurs with increased involvement (including Mental Support); this co-occurs with higher coparenting quality and more favorable resilience/adaptation profiles, ultimately relating to fewer depressive symptoms. In multiparous families, short leave had a small, supported negative total (indirect) association, whereas the total association of long leave was not clearly supported (its confidence interval included zero). This pattern may reflect limited power and/or heterogeneity in how leave time is translated into specific relational-support behaviors that most strongly predict coparenting (notably Mental Support), even though leave was associated with increased housework and some task-based involvement. Overall, these results suggest that leave duration alone may be insufficient; whether fathers’ leave results in increases in psychologically supportive, coordinating forms of involvement may be more consequential for coparenting quality.

### 4.4 Policy and perinatal practice implications

Our study has three main implications. First, policy evaluation should focus on leave uptake and duration and whether leave fosters the types of paternal involvement that matter for coparenting—especially Mental Support. Longer leave was associated with broader involvement in primiparous families, including Mental Support. Furthermore, this pattern corresponded to clearer indirect associations with lower maternal depressive symptoms. In multiparous families, leave was more strongly associated with task-oriented domains (especially Housework) and less clearly with Mental Support, which may explain the weaker and less consistent downstream associations. Therefore, policies and workplace supports may be most effective when they enable time off and conditions for meaningful family engagement (e.g., parenting confidence, shared planning, reduced isolation) during leave (Petts and Knoester, 2020; Petts et al., 2020). Evidence that fathers’ leave can be followed by sustained increases in their participation in childcare and housework further highlights the potential for longer-term shifts in unpaid work when conditions actively support leave utilization (Bünning, 2015). Policy implementation should also account for heterogeneity in leave experiences, workplace support, self-selection, and fathers’ adjustment during leave (Pizarro and Gartzia, 2024; Schaber et al., 2021; Terada et al., 2022).

Second, perinatal mental health services should incorporate couple-based content targeting coparenting processes and the “invisible” components of support. Considering the strong association of Mental Support with coparenting, interventions that teach concrete supportive behaviors (e.g., anticipatory support, shared decision-making, emotional validation, mental-load sharing) may be especially valuable. Studies revealed that family- and couple-based prevention and father-inclusive programs improved coparenting and reduced perinatal depressive symptoms (He et al., 2023; Takeishi et al., 2019; Tandon et al., 2021). Thus, addressing fathers’ own adjustment may also be important considering the associations between paternal postpartum depression, coparenting, and father–infant bonding (Wells et al., 2023; Wells and Jeon, 2023).

Third, supports may need to consider parity-related family contexts. Emphasis on building shared parenting schemas, supportive communication, and early coordination may be especially relevant for primiparous couples. For multiparous families, supports may need to extend beyond coparenting skills and include practical and structural resources, such as housework support, childcare for older children, and attention to financial strain; in the multiparous model, depressive symptoms were associated with individual vulnerability (D4) and perceived economic conditions in this group. This aligns with ecological perspectives emphasizing multi-level supports for parental adaptation, work–family adjustment, and parental burnout (Ahn et al., 2021; Ren et al., 2024).

The magnitude of the total indirect associations was small; therefore, these findings should not be interpreted as evidence that paternity leave alone produces clinically meaningful reductions in depressive symptoms at the individual level. However, small associations may still be relevant from a population and prevention perspective when they involve modifiable family-level processes, such as fathers’ supportive involvement and coparenting quality. These findings suggest that interventions should focus not only on promoting leave uptake but also on helping fathers use leave for emotionally supportive and coordinating involvement.

### 4.5 Limitations and future directions

Several limitations warrant consideration. First, this study’s cross-sectional design precludes causal inference; reverse causality and unmeasured confounding remain possible. Residual confounding also cannot be ruled out; unmeasured or insufficiently modeled demographic, occupational, relational, and family-related factors—such as education, work hours, workplace flexibility, prior relationship quality, paternal mental health, detailed infant health/temperament characteristics, and formal or informal support—may have influenced both fathers’ leave-taking and maternal outcomes. Furthermore, longitudinal designs with repeated measurement of involvement, coparenting, and maternal outcomes are required to assess temporal ordering and reciprocal processes (Wells et al., 2023; Wells and Jeon, 2023). In addition, because cross-group equality constraints were not imposed, the multi-group SEM provided parity-stratified estimates but did not formally test whether the structural associations differed by parity. Accordingly, differences in coefficient magnitude or in whether the 95% confidence intervals excluded zero should not be interpreted as evidence of statistically significant between-group differences. In particular, support for a path in one parity group but not in the other does not itself establish that the corresponding coefficients differed significantly. Future studies with *a priori* moderation hypotheses and sample-size planning specifically designed for cross-group comparisons should formally test equality constraints. Second, all measures of paternal involvement and coparenting were based on mothers’ reports. Although mothers’ perceptions may be particularly relevant to their own parenting adaptation and depressive symptoms, use of a single informant may have introduced shared-method variance, perceptual bias, or mood-congruent reporting. Mothers with more depressive symptoms may, for example, have rated fathers’ involvement and coparenting less favorably, which could have strengthened associations among maternally reported variables. The findings therefore should not be interpreted as objective assessments of fathers’ behavior or as evidence of agreement between mothers’ and fathers’ perspectives. Future studies should obtain reports from both parents and, where possible, include observational measures of father involvement and coparenting (Cabrera et al., 2018). Third, the father-involvement subscales showed moderate-to-strong intercorrelations; consequently, unique path estimates should be interpreted as domain-specific contributions conditional on the other domains and may be sensitive to measurement overlap. Fourth, we excluded CPRA D1 based on reliability and distributional checks in the current sample; therefore, this data-informed decision should be considered when interpreting the findings. Fifth, since the survey was conducted at 3 months postpartum, fathers in the short-leave group had returned to work during the assessment period, which may have attenuated or altered observed associations. Future longitudinal studies should incorporate detailed leave timing (start/end dates) and repeated measurements pre- and post-leave. Sixth, the recruitment and online survey procedures may have introduced selection and measurement biases. The sample was recruited through an online research panel and a company newsletter, and the analytic sample was limited to eligible mothers who completed the online survey, cohabited with the child’s father, and had no self-reported history of mental illness. The use of forced answering eliminated item-level missingness among survey completers but may have increased psychological reactance, survey dropout, or reduced response quality. Consequently, the analytic sample may overrepresent mothers who were willing to complete all required items, and some responses may have been influenced by the forced-answer format. The direction and magnitude of these potential selection and measurement biases could not be determined (Sischka et al., 2022). Therefore, the findings may not generalize to mothers without internet access, eligible mothers who did not complete the survey, or non-cohabiting families. Finally, because a reported diagnosis of postpartum depression would have led to exclusion under the depression category, the findings may not generalize to mothers with diagnosed postpartum depression, and the screening procedure may have restricted variability in depressive symptom severity. Conversely, postpartum depression was not assessed as a separate diagnostic category, and no clinical diagnostic interview was conducted; therefore, mothers with undiagnosed or unreported postpartum depression or elevated depressive symptoms may have remained in the sample. This self-reported screening procedure may have introduced diagnostic misclassification.

## 5 Conclusion

This study found that fathers’ paternity leave duration showed small indirect associations with mothers’ parenting adaptation and depressive symptoms at 3 months postpartum through fathers’ day-to-day involvement and coparenting quality. In primiparous families, both short and long leave were associated with broader paternal involvement and small negative indirect associations with depressive symptoms. In multiparous families, leave was mainly associated with housework and selected task-based involvement, and only short leave showed a small negative indirect association with depressive symptoms. Across parity groups, fathers’ mental support was the paternal involvement domain most strongly associated with coparenting quality. These findings suggest that paternity leave duration alone may be insufficient; the potential value of leave may depend partly on whether leave is accompanied by psychologically supportive involvement and stronger coparenting. Because the study was cross-sectional, these findings should be interpreted as theoretical associations rather than causal effects.

## Data Availability

The datasets presented in this article are not readily available because they contain sensitive personal information. Requests to access the datasets should be directed to the corresponding author.

## Appendix

**Table A1:** Model fit indices for the initial theory-specified and final refined multi-group SEM models

Model	Residual covariances	*χ*²(df)	*χ*²/df	CFI	TLI	RMSEA [90% CI]	PCLOSE
Initial theory-specified model	Not included	1098.181 (84)	13.074	0.644	−0.016	0.148 [0.140, 0.156]	<0.001
Final refined model	Included	133.216 (68)	1.959	0.977	0.919	0.042 [0.031, 0.052]	0.904

The initial model retained all hypothesized structural paths and included covariances among exogenous variables, but did not include residual covariances among endogenous residuals. The final refined model retained all structural paths and added a limited number of residual covariances among conceptually related subdomains measured by the same instrument. No structural paths were deleted or added *post hoc*.
